# Estimating levels of HIV testing coverage and use in prevention of mother-to-child transmission among women of reproductive age in Zambia

**DOI:** 10.1186/s13690-018-0325-x

**Published:** 2018-12-29

**Authors:** Brian Muyunda, Paul Mee, Jim Todd, Patrick Musonda, Charles Michelo

**Affiliations:** 1 Department of Epidemiology & Biostatistics, The University of Zambia School of Public Health, Lusaka, Zambia; 2 Ministry of Health, University Teaching Hospital, P/Bag RW 1X, 10101 Lusaka, Zambia; 3 London School of Hygiene and Tropical Medicine, Faculty of Epidemiology and Public Health, London, UK

**Keywords:** Prevention of mother to child transmission (PMTCT), Child bearing women, HIV, Zambia

## Abstract

**Background:**

Mother to child transmission of HIV (MTCT) still remains a challenge affecting many countries. Globally, an estimated 150,000 children were newly infected with HIV in 2015, over 90% of them in Sub-Saharan Africa through MTCT. In Zambia approximately 500,000 babies are born and 40,000 acquire the infection vertically if there is no intervention annually. This study estimated the HIV testing coverage and associated factors among Zambian women of reproductive age 15–49 years.

**Methods:**

A cross-sectional study based on data extracted from the Zambia Demographics and Health Survey [Zambia Demographic and Health Survey. Central Statistical Office (CSO), Ministry of Health (MOH), Tropical Diseases Research Centre (TDRC), University of Zambia, and Macro International Inc. 2009. 2014]. Women aged 15–49 years, 15,388 who reported having ever tested for HIV or not comprised the de facto eligible sample. Extracted data comprised women’s demographic characteristics; their full birth history and records of antenatal care for the most recent birth within a 5 year period preceding the survey. A weighted multiple logistic regression model was done to determine factors associated with the odds of HIV testing coverage among women of reproductive age.

**Results:**

Out of 15,388 women in the study, 12,413 (81%) reported ever tested for HIV. Of the 6461 women who attended antenatal care (ANC) 6139 (95%) reported ever tested for HIV. Additionally, 6139 (95%) out of 6461 of the women were given information on PMTCT during ANC sessions. Testing coverage was higher among women aged 20–24 years compared to women aged 15–19 years [AOR 2.1, 95% CI 1.14–3.84; *p* = 0.017]. Women with higher socio-economic status had 6.6 times the odds of having ever tested compared to women with lower status [AOR 6.6, 95% CI 3.04–14.14; *p* < 0.001].

**Conclusions:**

In this study we have demonstrated that HIV testing coverage is higher among women of reproductive age. HIV testing among women attending ANC is also higher. Older women with higher socio-economic status are more likely to take up HIV testing compared to their young counterparts.

## Background

The HIV/AIDS pandemic has been a devastating public health problem in Zambia and has drastically hindered the Country’s economic development especially among young productive women. Generally, many women especially the young engage in high risk activities such as cohabiting, multiple and concurrent sexual partners and low condom use. Many of them are victims of gender and sexual violence. At the same time, voluntary counselling and testing for HIV has been consistently low over the years. Most of the women choose not to test for fear of the positive results, stigma and discrimination ([1] Report; [2] Report). A similar pattern is observed among pregnant women.

Mother to child transmission of HIV (MTCT) is still a challenge affecting many countries. Globally, an estimated 150,000 children were newly infected with HIV in 2015, over 90% of them in Sub-Saharan Africa through MTCT [3]. Between 2012 and 2014, about 240,000 new-borns were newly infected with HIV, 90% of them in Sub-Saharan Africa (Wang, Q. et al., 2016; Perez et al., 2014). Without Prevention of Mother to Child Transmission (PMTCT), it has been reported that 15–30% of babies born to HIV positive women are infected during pregnancy and delivery, and a further 5–20% through breastfeeding (Obai et al., 2016; Kameel Mungrue, 2017). In resource constrained countries, approximately one third of HIV infected infants die before reaching 1 year and more than half die before reaching 2 years of age [4–8].

The consolidated guidelines for treatment and prevention of HIV infection in the PMTCT Program demands that medical practitioners adhere closely to the ANC protocol for better outcomes. The PMTCT package includes interventions provided to women during pregnancy such as maternal education, Prevention and treatment of STIs, Malaria (IPT), ultra-sound scans for foetal defect detection and HIV testing, prevention and treatment. A PMTCT cascade is a step by step successive decrease in the uptake of HIV services in the provision of PMTCT interventions. These steps include ANC utilization, Pre-test counselling, HIV test acceptance, receiving HIV test results and post-counselling, antiretroviral ARV prophylaxis to baby, intervention during labour and delivery and postnatal follow-up of mother and infant through 12–18 months of life and enrolment in care and support ([9–15]; Kameel Mungrue, 2017). For instance, one study in Nigeria observed that out of 31,504 women who entered PMTCT care during antenatal period, 20,679 (60%) completed the entire cascade of services (Holly E, R, et al., 2015).

Making PMTCT services accessible is essential for the elimination of infant HIV transmission in Sub-Saharan Africa. However, it was observed in a number of studies that PMTCT services had poor coverage and not accessible. For instance in a study in Ethiopia, although voluntary testing was provided freely, there were progressive and unacceptable losses to follow-up of 55, 68, and 70% of HIV-positive mothers during the antenatal period, delivery, and first postnatal visits, respectively and many deliveries occurred at peripheral sites where PMTCT was not available [16–24].

Another challenge in PMTCT coverage is acceptability of HIV services, which many developing countries with high HIV-prevalence face. Many pregnant women even after being diagnosed with HIV, there is no guarantee that they will accept treatment and adhere to it. For instance a study conducted in Burkina Faso and Côte d’Ivoire in West Africa observed that 40–60% HIV positive women did not accept any prophylaxis in pregnancy (Painter et al., 2004). Similarly, one cross-sectional study conducted in 26 communities of Zambia, South Africa, Cote d’Ivoire and Cameroon, observed that of the 976 women only 36% (355) completed every step of the PMTCT cascade and out of the remaining 621, 4% (22) did not seek antenatal care. Additionally, 17% (103) were not tested for HIV while 64% (395) had an HIV test, but did not receive their HIV positive results (Chi et al., 2015).

Zambia embarked on a PMTCT programme since 1999 to reduce the vertical transmission of HIV/AIDS which occur during pregnancy, labour, delivery and breastfeeding (MOT, 2009; WHO -PMTCT vision 2010–2015; MOH Zambia 2008). However, Zambia has a high rate of 45 infant deaths and 75 under five deaths per 1000 live births due to HIV related conditions [1]. Additionally, annually approximately 500,000 babies are born in Zambia and 40,000 acquire the infection vertically if there is no intervention [10]. In 2008, Zambia reported an antenatal HIV prevalence of 16.4% putting 80,000 infants at risk of getting infected with HIV/AIDS through Mother to Child Transmission ([25]; WHO -PMTCT vision 2010–2015; MOT, 2009). Another study showed that about 10% of all new HIV infections are attributable to MTCT [7]. Provision of HIV testing services to pregnant women is one of the key component in PMTCT programme and there is little published information on the coverage of HIV testing among women of reproductive age. This study estimated the HIV testing coverage and associated factors among Zambian women of reproductive age 15–49 years.

## Methods

### The 2014 ZDHS design

The data stem from Zambia Demographic and health Survey [1] conducted in 2014. The Zambia 2010 Population and Housing Census mapping system was used to establish the sampling frame (Census 2010 Report). The frame comprised 25,631 Enumeration Areas (EAs) or clusters. Firstly, 722 clusters were selected with probability proportional to size. At second stage, using equal probability systematic sampling, 18,052 households were drawn for the survey. The survey included a representative sample of 16,411 women aged 15–49 years. The detailed methods and key findings of the ZDHS 2014 have been reported elsewhere ([1] Report; Census 2010 Report). The datasets are available, on request, from the Zambia Central Statistical Office (CSO) and Ministry of Health (MOH), and also available through the website on demand from the Measure Consortium. (https://www.dhsprogram.com/).

A cross-sectional study design based on 2014 ZDHS women and HIV data was used for this paper. From the 16,411 women captured in the survey, 15,388 women who reported having tested for HIV or not in the ZDHS comprised the de facto eligible sample. The extracted de facto eligible sample included information on the women’s demographic characteristics; their full birth history and records of antenatal care for the most recent birth within a 5 year period preceding the survey including HIV testing (through dried blood sample collection and testing for the women eligible for the interview).

In this paper, we analysed HIV testing data from the women’s questionnaire. We used a binary outcome variable to indicate whether or not the respondent reported ever tested for HIV (Yes/No). Explanatory variables included The variables in the multiple logistic regression model women’s demographics such as age, marital status (Never in union, Married/living together, divorced/separated, widowed), socio-economic status (lower, medium, higher), as a proxy for Wealth tertile, residential area (urban or rural), place of delivery (home, Government hospital, other/private), and for those who reported attended ANC, ANC included PMTCT (Yes/No), ANC included HIV test (Yes/No).

### Statistical analysis

Both descriptive and inferential statistics were used to examine the HIV testing coverage in PMTCT and its associated factors. Univariate analysis (initially by cross tabulations using Pearson’s chi squared test) and later weighted multivariable logistic regression were performed to examine uptake of HIV services in PMTCT programme and control for any confounding. Considering this was clustered data, survey weights incorporating women’s individual sample weights, primary sampling unit (PSU) and sample strata for sampling errors were employed to obtain unbiased estimates for population parameters. A *p*-value of < 0.05 was taken as significant with 95% confidence intervals. Furthermore, in the statistical analysis, a step-wise multivariable regression was used as criteria for variable selection in the final model. The distribution of age as a continuous variable was adjusted for and conformed to normality as assessed by probability plots. We assessed interactions between wealth index and place of delivery; age and socio-economic status as well as place of delivery and whether ANC included HIV test or not. We also assessed collinearity and correlation between the variable whether ANC included HIV test or not and ANC covered PMTCT. The variables in the multiple logistic regression model were age, residence (urban or rural), marital status, socio-economic status), ANC attendance, partner age as well as maternal and place of delivery information (categorized as public, private, home or other facility). Stata version 14.0 was used for all analyses (Statacorp).

### Ethics

The ZDHS 2014 obtained ethical approval from Tropical Diseases Research Centre (TDRC) in Ndola Zambia and the US Centres for Disease Control and Prevention (CDC) Atlanta Research Ethics Review Board ([1] Report). Voluntary informed consent was obtained for all participants in DHS. Data were anonymised and made available for public use. Therefore, reanalysis of the data did not interfere with participant’s privacy and posed minimal to no risk. Additionally, a waiver was obtained from the University of Zambia Biomedical Research Ethics Committee (UNZABREC) and granted permission to conduct this study under the Sustainable Evaluation and Analysis of Routinely Collected HIV data (Ref. no. 010–04-16).

## Results

### Participation and distribution

**Fig. 1:**
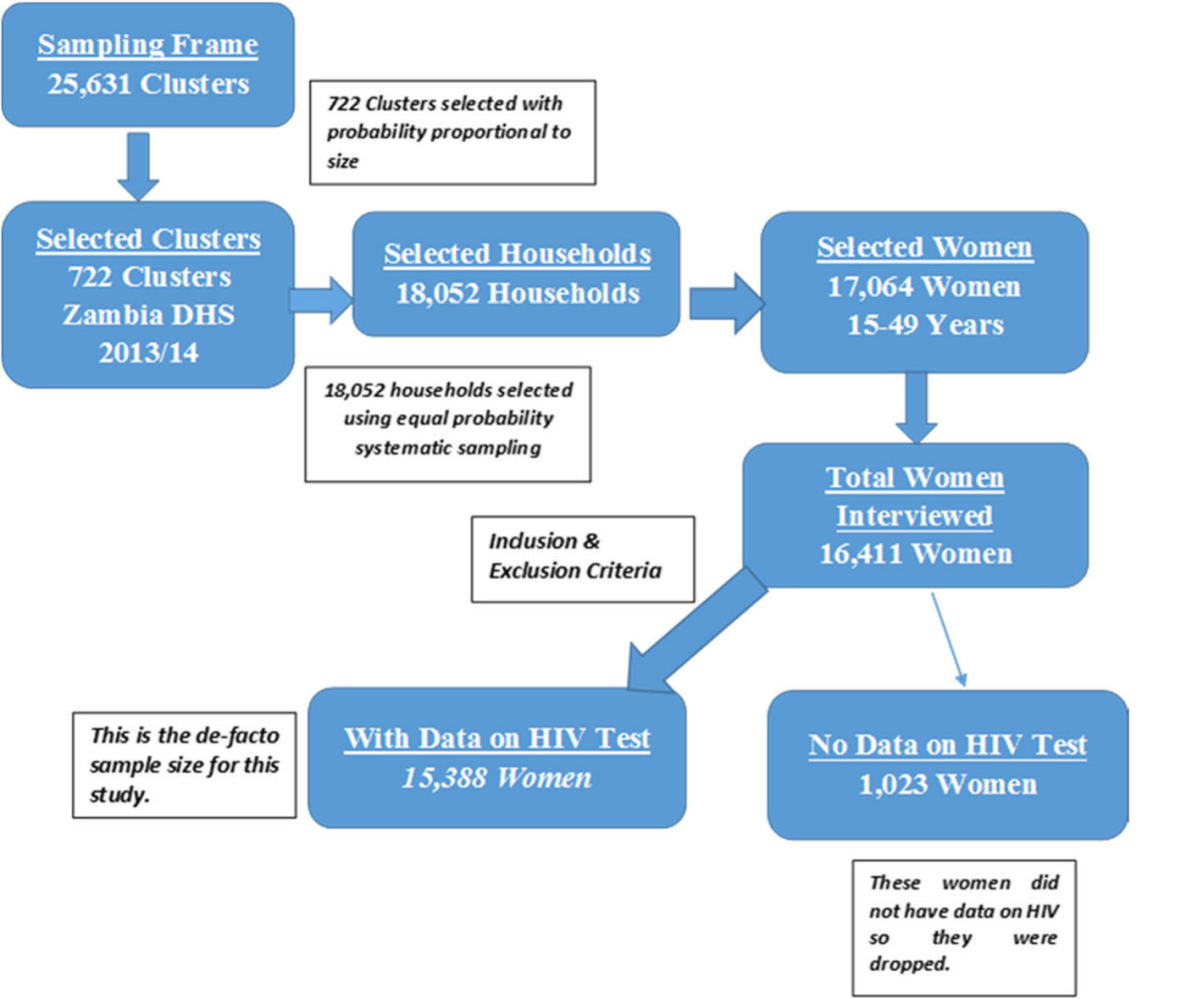
Sampling design for the study based on 2014 ZDHS, on HIV testing coverage among women aged 15–49 years

Of the 16,411 women aged 15 to 49 years, in the households selected for ZDHS, a total of 15,388 women had data on their HIV testing (Fig. 1). Of the 15,388 women, the median age in years was 27 (IQR = 23 to 32) with 4901 (32%) women in the age range 25–34 years. 9101 (59%) of the women were married and 47% (7316) women living in urban areas (Table 1).

**Table 1:** Social Demographics and Other Characteristics of 15,388 women aged 15–49 years in Zambia with data on HIV testing

Characteristics	Frequency	(Percent)	95% CI
Age group (in Years)			
15–19	3487	22.6	[21.9, 23.3]
20–24	2888	18.7	[18.1, 19.3]
25–34	4901	31.8	[31.0, 32.5]
35–49	4157	26.9	[26.2, 27.6]
Residence			
urban	7316	47.4	[46.6, 48.2]
rural	8117	52.6	[51.8, 53.4]
Marital Status			
Not Married	4456	28.9	[28.2, 29.6]
Married/Living together	9101	59.0	[58.2, 59.7]
Widowed/Divorced	1553	10.1	[9.6, 10.5]
Separated	323	2.0	[1.9, 2.3]
Socio-economic Status			
Lower	5551	36.0	[35.2, 36.7]
Medium	3328	21.6	[20.9, 22.2]
Higher	6554	42.4	[41.7, 43.2]
Received Results after HIV test			
No	207	1.7	[1.5, 1.9]
Yes	12,100	98.3	[98.1, 98.5]
Mode of HIV test			
Client Initiated testing	4698	51.6	[50.6, 52.6]
Provider Initiated testing	3039	33.4	[32.4, 34.4]
Other	1367	15.0	[14.3, 15,8]
Place of Delivery			
Home	2373	26.8	[25.9, 27.7]
Government Facility	5921	66.8	[65.8, 67.8]
Other	569	6.4	[5.8, 6.9]
ANC Included PMTCT			
No	399	6.1	[5.6, 6.7]
Yes	6133	93.9	[93.3, 94.4]
HIV test Offered in ANC			
No	313	4.8	[4.3, 5.4]
Yes	6198	95.2	[94.6, 95.7]

### HIV testing coverage during ANC

Out of the total of 15,388 women in the study, 12,413 (80.6%) reported ever tested for HIV in the ZDHS. Of those who tested for HIV, 12,100/12,413 (98%) received their HIV test results. (Table 2).

**Table 2:** Social background Characteristics of 15,388 women aged 15–49 years in Zambia with data on HIV testing

*N* = 15,388	Ever Tested for HIV					95% CI	*P*-Value
Characteristics	No			Yes			
	n	(%)	95% CI	n	(%)		
Age group (in Years)							**< 0.001** ^b^
15–19	1723	50.6	[48.1, 53.1]	1681	49.4	[46.9, 51.8]	
20–24	318	11.2	[9.7, 12.8]	2530	88.8	[87.2, 90.3]	
25–34	334	6.8	[5.9, 7.9]	4561	93.2	[92.1, 94.1]	
35–49	582	14	[12.7, 15.5]	3560	86	[84.5, 87.3]	
Residence							**0.023**
urban	1258	18.1	[16.7, 19.6]	5694	81.9	[80.4, 83.3]	
rural	1699	20.4	[19.1, 21.7]	6639	79.6	[78.3, 80.9]	
Marital Status							**< 0.001**
Not Married	1941	45.7	[43.4, 47.9]	2310	54.3	[52.1, 56.6]	
Married/Living together	817	8.9	[7.9, 9.9]	8406	91.1	[90.1, 92.1]	
Widowed	174	11.7	[10.0, 13.7]	1316	88.3	[86.3, 90.0]	
Divorced	25	7.6	[4.7, 12.0]	301	92.4	[88.0, 95.3]	
Socio-economic Status							**0.022**
Lower	1115	20.7	[19.1, 22.3]	4279	79.3	[77.7, 80.9]	
Medium	504	17.3	[15.7, 18.9]	2409	82.7	[81.0, 84.3]	
Higher	1338	19.2	[17.8, 20.6]	5645	80.8	[79.4, 82.2]	
Place of Delivery							**< 0.001**
Home	296	12.4	[10.4, 14.7]	2091	87.6	[85.3, 89.6]	
Government Facility	187	3.2	[2.6, 3.9]	5656	96.8	[96.1, 97.4]	
Other	16	3.3	[−]	498	96.7	[−]^q^	
ANC Included PMTCT							**< 0.001**
No	91	22.8	[17.8, 28.7]	310	77.2	[71.3, 82.2]	
Yes	156	2.6	[2.0, 3.2]	5922	97.4	[96.8, 98.0]	
HIV test Offered in ANC							**< 0.001**
No	203	63.8	[−]	115	36.2	[−]	
Yes	44	0.7	[0.5, 1.0]	6095	99.3	[98.9, 99.5]	

Note:

*The total sample size reduced (from *n* = 15,388 to 9104), because some variables had missing values and were excluded in the analysis and also non-participation due to skip patterns on some questions

b. Pearson Chi-squared test was used to obtain *p*-values. This applies for all *p* values

q. Missing Confidence intervals because of missing standard errors due to stratum with single sampling unit

During ANC, 6139/ 6461 (95%) of women reported ever tested for HIV. Additionally, 6139/6461 (95%) women were given information on HIV and PMTCT during ANC sessions. (Table 3).

**Table 3:** Background Characteristics of 6461 Women aged 15–49 years Reported on HIV Testing during ANC

*N* = 6461	Tested for HIV in ANC					95% CI	*P*-Value
Characteristics	No		95% CI	Yes			
	n	(%)		n	(%)		
Age group (in Years)							0.336
15–19	44	5.7	[4.0, 8.1]	719	94.3	[91.9, 95.8]	
20–24	75	4.6	[3.3, 6.3]	1564	95.4	[93.7, 96.7]	
25–34	131	4.6	[3.7, 5.8]	2714	95.4	[94.2, 96.3]	
35–49	72	5.9	[4.5, 7.7]	1142	94.1	[92.3, 95.5]	
Residence							< 0.001
urban	35	1.6	[1.1, 2.3]	2174	98.4	[97.7, 98.9]	
rural	287	6.8	[5.6, 8.2]	3965	93.2	[91.8, 94.4]	
Marital Status							0.348
Not Married	24	3.6	[2.3, 5.6]	638	96.4	[94.4, 97.7]	
Married/Living together	264	5.1	[4.2, 6.1]	4954	94.9	[93.9, 95.8]	
Widowed	23	5.6	[3.5, 8.6]	389	94.4	[91.4, 96.5]	
Socio-economic Status							< 0.001
Lower	228	7.6	[−]^q^	2785	92.4	[−]^q^	
Medium	56	4.1	[3.0, 5.6]	1304	95.9	[94.4, 97.0]	
Higher	38	1.8	[1.2, 2.7]	2050	98.2	[97.3, 98.8]	
Place of Delivery							< 0.001
Home	169	9.9	[−]	1548	90.1	[−]	
Government Facility	140	3.2	[3.0, 5.6]	4184	96.8	[94.4, 97.0]	
Other	13	1.8	[1.2, 2.7]	406	98.2	[97.3, 98.8]	
ANC Covered PMTCT							< 0.001
No	125	31.0	[25.1,37.6]	277	69.0	[62.4,74.9]	
Yes	197	3.3	[2.6,3.9]	5862	96.7	[96.0,97.4]	
Mode of HIV test							0.253
Client Initiated testing	29	2.3	[1.5, 3.5]	1215	97.7	[96.5, 98.5]	
Provider Initiated testing	35	3.0	[2.2, 4.4]	1142	97.0	[95.6, 97.9]	
Other	20	3.9	[2.4, 6.4]	475	96.1	[93.6, 97.6]	

*Note:*

b. Pearson Chi-squared test was used to obtain *p*-values. This applies for all *p* values

q. Missing Confidence intervals because of missing standard errors due to stratum with single sampling unit

### Determinants of HIV testing

Testing coverage was higher among women aged 20–24 years compared to women aged 15–19 years [AOR 2.1, 95% CI 1.14–3.84; *p* = 0.017]. Additionally, HIV testing uptake had significantly lower levels among rural women with 80% reduced odds compared to urban women [OR 0.2, 95% CI 0.1–0.6; *p* = 0.003] though after adjusting for age and other variables this was not significant (Table 4).

**Table 4:** Key Predictors of HIV Testing Among women aged 15–49 years who participated in the Demographic and Health survey 2014- from Multiple Logistic Regression Analysis

N = 15,433	Category	OR	Crude	*P*-Value	AOR	Adjusted	*P*-Value
Characteristic			95% CI			95% CI	
Age group (In Years)	15–19	Ref			Ref		
	20–24	8.1	[6.9–9.7]	< 0.001	2.1	[1.1–3.9]	0.017
	25–34	13.9	[11.6–16.8]	< 0.001	2.0	[1.1–3.4]	0.034
	35–49	6.3	[5.4–7.3]	< 0.001	1.4	[0.8–2.7]	0.286
Marital Status	Single	Ref					
	Married	8.6	[7.5–10.0]	< 0.001			
	Widowed/Divorced	6.3	[5.2–7.8]	< 0.001			
	Separated	10.2	[6.1–16.9]	< 0.001			
Socio-economic Status	Lower	Ref			Ref		
	Medium	1.2	[1.09–1.43]	0.001	1.6	[0.9–2.6]	0.101
	Higher	1.0	[0.96–1.25]	0.162	6.6	[3.1–14.5]	< 0.001
Residence	Urban	Ref					
	Rural	0.8	[0.76–0.98]	0.023			
Place of Delivery	Home	Ref					
	Government Facility	4.3	[3.29–5.54]	< 0.001			
	Other	4.2	[2.36–7.48]	< 0.001			
ANC covered HIV PMTCT	No	Ref					
	Yes	12.0	[8.39–17.19]	< 0.001			
ANC Included HIV test	No	Ref			Ref		
	Yes	245.2	[159.16–377.74]	< 0.001	100	[59.2–172.1]	< 0.001
Interaction terms							
Delivery Place ANC included HIV test	Facility **vs** Yes				4.7	[2.0, 11.2]	< 0.001
	Other **vs** Yes				9.5	[0.9, 97.6]	0.06

Testing coverage was higher among women aged 20–24 years compared to women aged 15–19 years [AOR 2.1, 95% CI 1.14–3.84; *p* = 0.017]. Women with higher socio-economic status had 6.6 times the odds of having ever tested compared to women with lower status [95% CI 3.04–14.14; *p* < 0.001].

Furthermore, women with a higher socio-economic status had 6.6 times the odds of having ever tested compared to women with a lower status [AOR 6.6, 95% CI 3.04–14.14; *p* < 0.001]. Higher HIV testing coverage among women who attended ANC and delivered from the facility whether Government or private compared to those who delivered from home [AOR 6.2, 95% CI 1.68–22.53; *p* = 0.006]. The interaction of place of delivery and whether ANC included HIV testing had a strong positive effect on the odds of HIV testing uptake [AOR 4.7; 95% CI 2.0–11.2; *P* < 0.001]. In the multi-variable analysis, age, socio-economic status of woman and ANC included HIV test were the only covariates that were statistically significant while marital status, residence, and ANC covered PMTCT were not (Table 4).

The assessed interactions between socio-economic status and place of delivery; age and socio-economic status; as well as place of delivery and ANC included HIV testing were considered not important because they were not statistically significant. However, the interaction between Place of delivery (facility) and ANC including HIV testing had a combined positive effect on HIV testing coverage [AOR 4.7, 95% CI 2.0–11.2; *P* < 0.001]. The collinearity assessment between the independent variables ANC included HIV and ANC covered PMTCT showed that both the regression analysis tolerance (0.890) and the variance inflated factor (1.11) were close to 1 implying that the variables were either uncorrelated or had a weak correlation.

## Discussion

In this study we observed an overall increase in reported uptake of HIV testing among child bearing women from 60% in 2009 to over 80% in 2014 ([1] Report; [2] Report). Generally, many women especially the young engage in high risk activities such as cohabiting, multiple and concurrent sexual partners and low condom use. Many of them are victims of gender and sexual violence. At the same time, voluntary counselling and testing for HIV has been consistently low over the years. Most of the women choose not to test for fear of the positive results, stigma and discrimination. Similar results were observed among pregnant women attending ANC.

Universal access to HIV testing as a gateway to prevention, care, support and treatment is an important objective of national PMTCT programme Having a single test during pregnancy however, may be risky for the unborn child. Any pregnant woman is expected to have a serological HIV test during first ANC and repeat the test every 3 months if negative. This means that every consecutive ANC trimester visit every pregnant woman is expected to have an HIV test (Zambia Consolidated Guidelines, 2016).

Although HIV testing services are offered at no cost to all women in ANC in a respectful setting that accords them autonomy whether to test or opt out, over 95% of the women had an HIV test and collected results. This finding could suggest the importance of counselling before and after HIV testing which may be taken for granted. The consolidated guidelines for HIV testing, care and treatment in Zambia stipulates that high quality pre-testing and post-testing counselling should be offered to all without discrimination (Zambia Consolidated HIV Guidelines, 2016). Possible explanation for this could be that when counselled, women may have a better understanding of HIV/AIDS related risks and transmission to the unborn child, relieved worries of being HIV positive for those testing negative, and referral to treatment for those testing HIV-positive and realising that their unborn child could be prevented from being infected.

Despite the fact that ANC attendance is over 90%, facility delivery is consistently low. Women who delivered from facilities were more likely to undergo HIV testing compared to those that delivered from home thus exposing their unborn babies to vertical transmission. Similar results were observed that failure to deliver from health facility reduces chances of women to utilize HIV testing services [26]. Many of these HIV positive women perhaps feared that if they delivered from the facility, their friends and relatives would know their HIV status and home delivery was considered a solution. Furthermore, in one study on utilization of PMTCT services and acceptability in Ethiopia showed that many women declined HIV testing in ANC due to poor understanding and fear of stigma (Meda et al., 2002, [27, 28]). This calls for innovative ways of reaching out to these women who fail to access facility HIV services such as home-based counselling and testing in the comfort of their homes.

Home-based HIV counselling and testing could be a better alternative for pregnant women who fail to access health facility based counselling and testing. In Zambia a robust cluster randomised trial was conducted to investigate the feasibility and acceptance of home-based HIV-testing. Findings showed that acceptance and uptake was about 5 times higher compared to clinical based testing. Similar findings have been reported in other countries in Sub-Saharan Africa where offering of home-based HIV testing and counselling has led to increased use [29–33].

Selection biases such as non-participation by not attending ANC and absence during the ZDHS survey could have possibly affected our findings and estimates. However, the magnitude of this effect can only be assessed to some extent like any other study with similar design. Participation in this survey was about 98% implying that the most absence was because of not attending ANC. However, we are very confident of our results because ANC attendance of at least one visit is over 95% and the number of self-reported pregnant women who did not attend ANC was negligible. Therefore, we are very confident that the non-participation was very minimal and not important in altering our findings.

Furthermore, like in most of secondary analysis-based studies, the data were not tailored with this objective as a focus and as such predictor measures may not capture exactly initial measures intended. It may not be possible to conclusively control for all confounding and interactions in these findings due to uncontrolled confounding effect that may result from other forms of non-participation bias such as misclassification of missing values which is a threat to most data. In this analysis, we controlled for the effect of confounding through stratification during the analysis process. Taking into account all these limitations, we still believe their effect is minimal and unimportant in explaining our findings. Hence these findings may help programme implementers and policy makers in channelling limited resources and interventions in particular areas of real need for better maternal and new-born health outcomes.

### Recommendations

This study identified a number of potential strategies and recommendations that can help reduce MTCT in Zambia. Strengthening HIV testing in rural health facilities, encouraging women to deliver in facilities and provide initiatives that seek to overcome barriers to testing among young mothers are some of the ways that can help improve maternal and child health. Obtaining and analysing routine health systems data is important to estimate the coverage of PMTCT programmes.

## Conclusions

In this study we have demonstrated that HIV testing coverage is high among child bearing women. Despite this study showing a high HIV test rate, this could be linked to the first ANC attendance considering that in Zambia, first ANC attendance is over 90% whilst adequate ANC utilization of recommended at least four visits is still limited. In order to experience the full benefits of HIV testing in the PMTCT programme, adequate ANC utilization with an HIV test at each consecutive ANC visit is crucial. Therefore, encouraging women to complete ANC and deliver from the health facilities is key in reducing vertical transmission.

## Abbreviations

ANC: Antenatal Care
ARV: Antiretroviral
CDC: Centres for Disease Control and Prevention
CSO: Central Statistical Office
EA: Enumeration Area
MOH: Ministry of Health
MTCT: Mother to Child Transmission
PMTCT: Prevention of mother to child transmission
PSU: Primary Sampling Unit
SEARCH: Sustainable Evaluation and Analysis of Routinely Collected HIV data
TDRC: Tropical Diseases Research Centre
UNZABREC: University of Zambia Biomedical Research Ethics Committee
WHO: World Health Organization
ZDHS: Zambia Demographics and Health Survey
